# Impact of T Lymphocytes Isolated from Liver Perfusate of Deceased Brain Donors on Kidney Transplantation: Preliminary Evidence and Future Directions

**DOI:** 10.3390/jcm12144786

**Published:** 2023-07-20

**Authors:** Duilio Pagano, Ester Badami, Giovanni Zito, Pier Giulio Conaldi, Ivan Vella, Barbara Buscemi, Giandomenico Amico, Rosalia Busà, Paola Salis, Sergio Li Petri, Fabrizio di Francesco, Sergio Calamia, Pasquale Bonsignore, Alessandro Tropea, Caterina Accardo, Salvatore Piazza, Salvatore Gruttadauria

**Affiliations:** 1 Abdominal Surgery and Organ Transplant Unit, Department for the Treatment and Study of Abdominal Diseases and Abdominal Transplantation, Instituto di Ricovero e Cura a Carattere Scientifico (IRCCS)-Mediterranean Institute for Transplantation and Advanced Specialized Therapies (ISMETT), University of Pittsburgh Medical Center Italy (UPMCI), 90127 Palermo, Italy; dpagano@ismett.edu (D.P.); slipetri@ismett.edu (S.L.P.); fdifrancesco@ismett.edu (F.d.F.); scalamia@ismett.edu (S.C.); pbonsignore@ismett.edu (P.B.); atropea@ismett.edu (A.T.); caccardo@ismett.edu (C.A.);; 2 Department of Laboratory Medicine and Advanced Biotechnologies, Instituto di Ricovero e Cura a Carattere Scientifico (IRCCS)-Mediterranean Institute for Transplantation and Advanced Specialized Therapies (ISMETT), 90127 Palermo, Italy; ebadami@fondazionerimed.com (E.B.); gzito@ismett.edu (G.Z.); pgconaldi@ismett.edu (P.G.C.); rbusa@ismett.edu (R.B.); 3 Ri.MED Foundation, 90133 Palermo, Italy; gamico@fondazionerimed.com; 4 Nephrology Unit, Department for the Treatment and Study of Abdominal Diseases and Abdominal Transplantation, Instituto di Ricovero e Cura a Carattere Scientifico (IRCCS)-Mediterranean Institute for Transplantation and Advanced Specialized Therapies (ISMETT), University of Pittsburgh Medical Center Italy (UPMCI), 90127 Palermo, Italy; bbuscemi@ismett.edu (B.B.); psalis@ismett.edu (P.S.); 5 Department of Surgery and Surgical and Medical Specialties, University of Catania, 95124 Catania, Italy

**Keywords:** kidney transplantation, hepatic perfusate, deceased brain donors, T lymphocytes

## Abstract

Background: Ischemia/reperfusion injury (IRI), acute rejection (AR), and delayed graft function (DGF) might occur as major complications following kidney transplantation. Thus, the identification of biomarkers for the IRI, AR, and/or DGF development becomes crucial as it may help to guide post-transplant management. Natural killer (NK) cells, hepatic interstitial T-lymphocytes (T-Li), and NK-T cells are crucial in both innate and adaptive immunity after abdominal solid organ transplantation. Hence, the aim of this study was to evaluate the impact of the immune system after graft reperfusion during KT in adults in order to identify predictive biomarkers. Methods: The NK, T-Li, and NK-T phenotypes and concentrations were retrospectively analyzed in a consecutive series of liver perfusates obtained after organ procurement flushing the abdominal cavity recovered from deceased brain donors (DBDs). Their percentage was compared with the renal transplant recipients’ characteristics with kidneys taken from the same DCDs. The hepatic perfusate cells were purified by density gradient centrifugation. Flow cytometric investigation was used to determine their phenotype with the following immunological markers in order to determine the relative percentage of T-Li, NK-T, and NK cells: CD3, CD4, CD8, and CD56. Results: 42 DBDs’ liver perfusates were analyzed. The related clinical outcomes of kidney transplant recipients from 2010 to 2020 performed at our Institute were evaluated. Time in days of delayed functional recovery of transplanted kidneys (DGF) (*p* = 0.02) and the onset of secondary infection from a cytomegalovirus (*p* = 0.03) were significantly associated with the T-Li percentage. An increased relative risk (HR) of organ survival was significantly associated with the percent cell concentration of T-Li and time to DGF, on COX analysis, were (HR = 1.038, *p* = 0.04; and HR = 1.029, *p* = 0.01, respectively). None relevant clinical outcomes in kidney transplant patients were associated with the specificity of the NK and NK-T cell proportions. Conclusions: A new potential role of T-Li cells was detected in the context of hepatic perfusate from DBDs. It could detect potential impacts in organ allocation, surgical procuring techniques, and in the analysis of IRI pathophysiological events.

## 1. Introduction

The treatment of choice for end-stage renal disease is kidney transplantation (KT). Its success depends on many factors, including the compatibility of the donor and recipient, the quality of the transplanted organ, and the immune response of the recipient [1]. Acute rejection (AR) and delayed graft function (DGF) are common complications following KT, which can have a negative impact on the long-term outcome of the allograft [2]. The identification of biomarkers for the AR and/or DGF development may help to guide post-transplant management and improve the long-term outcome of KT [3]. The perfusion fluid of donated organs contains a wealth of information about the immunologic and metabolic state of the organ and the extent of any damage that may have occurred due to ischemic insult or other insults [4]. The proportion of T lymphocyte (T-Li) has been suggested to play a role in the development of DGF and AR following deceased donor KT [5]. The mechanisms underlying this association are not fully understood, and recently, many studies have investigated the possible immune reaction suppression by modulating T-Li in the transplant organ to reduce the triggering of an immune response in the recipient, leading to DGF and AR [6]. In addition, studies of similar models of IR after KT have shown the associations between the proportion of donor T-Li and the deleterious potential of ischemia-reperfusion injuries following organ revascularization [7,8,9,10,11,12]. Therefore, we conducted a retrospective and monocentric study to evaluate its impact after graft reperfusion during KT in adults.

## 2. Materials and Methods

### 2.1. Donor Cohort and Liver Perfusate Fraction Analysis

T-Li, natural killer (NK), and NK-T cellular concentrations and phenotypes were retrospectively analyzed in a consecutive series of liver perfusates (LPs) after hepatectomy of whole livers on the bench recovered from deceased brain donors (DBDs). Leucocytes were isolated from the perfusion fluid of liver explants, as previously described [13,14,15]. Briefly, during the organ procurement, the aorta was clamped, and the abdominal cavity was flushed with up to 5 L of preservation solution to exsanguinate the deceased donor. Liver perfusate (LP) was collected via a vacuum-pump suction system directly into an autotransfusion reservoir (ATR) (Fresenius-Kabi, Bad Homburg, Germany), filled with the anticoagulant acid citrate dextrose solution (Fresenius-Kabi).

Their percentage was compared with the renal transplant recipients’ characteristics with kidneys taken from the same DCDs [13]. The liver interstitial cells were purified from the perfusate by density gradient centrifugation, and the phenotype was determined by flow cytometric investigation using the following immunological markers: CD3, CD4, CD8, and CD56.

T-Li, NK-T, and NK subpopulations were first gated according to their expression of CD3 to discriminate between T-Li (CD3+CD56-), NK-T (CD3+CD56+), and NK cells (CD3-CD56+). T-Li was then further classified according to the expression of surface CD4 (helper T cells, Th) or CD8 (cytotoxic T lymphocytes, CTLs). The controls used were peripheral blood mononuclear cells (PBMC). During the back-table surgical time and after the procurement procedures, LPs were collected in a series of DBDs for adult patients at IRRCS-ISMETT from 2010 to 2020. To render the entire study population homogenous, the following DBD conditions were excluded:

- Pregnancy or active breast-feeding,
- Allergy or autoimmune disease,
- Donors requiring systemic immunosuppressive drug at the time of procurement,
- History of malignancy and of human immunodeficiency virus positivity,
- Donor who had previously received an organ transplant.

BD Vacutainer^®^ Blood Collection Tube containing K2 ethylenediaminetetraacetic acid was used to collect donor peripheral blood samples (Becton, Dickinson and Company, Plymouth, UK). All of the graft procurement was performed at the same organ procurement with identical surgical instruments and skilled medical and nursing staff; standard pressure and temperature setting of perfusion were used, except for administering two different organ preservation solutions after cross-clamp maneuver: Celsior solution (SangStat Medical Corporation, Fremont, CA, USA) or University of Wisconsin (UW; ViaSpan, DuPont Pharmaceuticals, Wilmington, DE, USA) as published elsewhere [13].

### 2.2. Flow Cytometry Staining of Lymphocytes and Antibody

Aliquots of 1 × 10^6^ isolated liver-derived cells and PBMC were stained for surface markers with pre-diluted fluorochrome-conjugated anti-human monoclonal antibodies: BD Multitest™ CD16-PE (IgG1, clone B73.1) and CD56-PE (IgG1, clone NCAM 16.2); CD3/CD16+CD56/CD45/CD19 reagent (CD3-FITC (IgG2a, clone SK7); CD45-PerCP (IgG1, clone 2D1); CD335/NKp46-PE-Cy7 (IgG1, clone 9E2/Nkp46); CD19-APC (IgG1, clone SJ25C1)) and CD3-FITC (IgG2a, clone SK7); CD4-PE-Cy7 (IgG1, clone L200); CD56-AlexaFluor-700 (IgG1, clone B159); CD8-APC-Cy7 (IgG1, clone SK1); antiCD337/NKp30-AlexaFluor-647 (IgG1, clone p30-15); and CD314/NKG2D-PerCP-Cy5.5 (IgG1, clone 1D11). These antibodies were purchased from BD Biosciences (Europe, dilution 1:10). CD161-FITC (IgG2a, clone 191B8); CD3-PerCP (IgG2a, clone BW264/56); CD16-APC (IgM, clone VEP13); and CD336/NKp44-PE (IgG1, clone 2.29) from Miltenyi Biotec (Bergisch Gladbach, Germany, dilution 1:20). TCRVa7.2-PE (IgG1, clone 3C10) was obtained from Biolegend (Campoverde s.r.l., Milan, Italy, dilution 1:25). At room temperature in the dark, LP samples were incubated for 30 min, and washed once with PBS/2% FBS. They were analyzed by flow cytometry with a FACSAria II cytometer (BD Biosciences, Eysins, Switzerland). 

### 2.3. Recipient Cohort and Study Endpoints

All KT recipients with kidney grafts procured from deceased after circulatory death donors (DCDs) and DBDs were initially included. The following recipients were excluded:

- Dual KT and/or combined solid organ transplantation recipients,
- A recipient who underwent KT with a graft procured from a DBD affected with thrombotic micro-angiopathy,
- Recipients of a living donor transplant.

Routinely, the DCD kidney was preserved at 4 °C with hypothermic machine perfusion (HMP) (Kidney Assist^®^ Organ Assist Product, Groningen, The Netherlands), with a fixed systolic perfusion pressure of 25 mmHg, to face prolonged ischemia time (>20 h) as a result of extra-regional donors, while awaiting cross-match results and recipient preoperative dialysis [13]. The primary endpoint of this study was the incidence of delayed graft function (DGF). It was defined as:

- Acute kidney injury (AKI) needs dialysis within 1 week of transplantation [1];
- Incidence of early graft loss (EGL) at 6 months after KT.

Overall and intensive care unit (ICU) length of stay, operating room readmission, hospital readmission (within 30 days), biopsy-proven acute cellular, humoral, and vascular rejection episodes, and post-operative complications such as onset of secondary infection from a cytomegalovirus (CMV) were evaluated as secondary endpoints. 

### 2.4. Statistical Analysis

The T-Li median value of flow cytometric results was used to divide the LP population into two subgroups (inferior vs. equal or superior of the median ranks). Student t-test or the Wilcoxon rank-sum test were used to determine significant differences between continuous variables among these groups. Categorical variables were analyzed with the chi-square test or Fisher’s exact test, as appropriate. Multiple logistic regression was used to detect potential independent risk factors. Particularly, odds ratios (ORs) with the corresponding 95% confidence interval (95% CI) were calculated from those variables that were identified as statistically significant in group comparison tests. The following DBD variables were included in these analyses: gender, age, weight, height, body mass index (BMI), cause of death, hemodynamic risk factors such as amine for more than 6 h to sustain blood pressure for prolonged hypotension, and ICU length of stay, including use of cold ischemic time (CIT). Donor bacterial infections, viral serologic markers, history of diabetes or glucose intolerance, and laboratory exams as donor liver function tests (aspartate aminotransferase—AST, alanine aminotransferase—ALT, gamma-glutamyl transferase—GGT, total bilirubin), and natremia before aortic cross-clamp. Early graft-loss/donor risk index (EGL-DRI) was evaluated as a semi-continuous factor to consider a multifactorial scoring system to identify organs at the highest risk for DGF [16].

Linear regression analysis was performed for semi-continuous variables when significant univariate associations to the different percentages of cellular fractions were determined. Statistical significance was determined at *p* < 0.05.

### 2.5. Ethical Validation

In accordance with the Declaration of Helsinki of 1996 and the Institutional Research Review Board (IRRB) approval of the protocol (protocol number IRRB/14/15), the study was conducted.

## 3. Results

All livers were used for transplant purposes at ISMETT. Of the kidneys recovered (n = 84), 42 were transplanted by our center, while the remaining 42 were assigned to other centers in Sicily. The related KT recipients’ clinical outcomes and the LPs’ cellular percentages were analyzed. The demographic and clinical characteristics of the donors and recipients are described in Table 1.

The proportion of CD8+ CTLs was significantly increased compared to CD4+ T helper cells in the LPs. By contrast, the opposite ratio was observed in matched PBMC. The median percentages of T-Li were 25.0%, based on the entire lymphocytic population obtained by cytofluorimetric analyses. 

The two different organ preservation solutions have not influenced any discrepancies in terms of T-Li, NK, and NKT cellular percentages isolated in the LPs. Neither history of viral infection, cardiac arrest, amine infusion, diabetes, nor glucose intolerance had any correlation with different lymphocyte LP proportions. CD3+CD56- T lymphocytes < 25% had a percent of CD8+ T-cells as high as 88.9% of total T-cells from LPs. CD8+ T cells percent of total T-cells in the perfusate was lower (64.7%) among DBDs, with more than 25% of perfusate leucocytes being CD3+ T cells. A significant association between low T-cell proportion and cold ischemia time was found. The average cold ischemia time was 522 ± 443.6 min in DBD liver perfusate with a T-cell percentage below 25% of total cells (*t*-test, *p* = 0.03) (Table 2).

**Figure 1:**
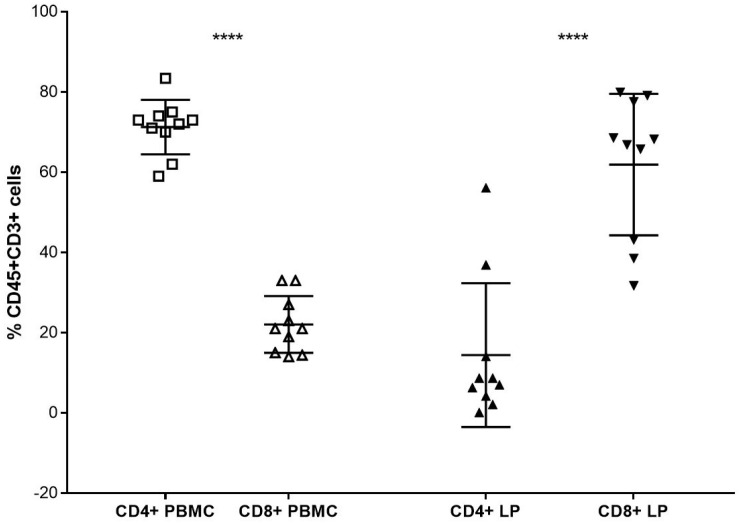
**Phenotype of T cells in liver perfusate.** CD8+ cytotoxic T lymphocytes were highly represented in the back-table fraction of the liver perfusate of matched donors and significantly more abundant compared to CD4+ T cells (**** *p* < 0.0001).

Ex situ LPs were collected during the pre-transplantation albumin perfusion of 46 DBD livers. The mean CIT was 481 ± 122.8 min and the average donor age was 52 ± 19.6 years. There were 24 female donors, and the main cause of death was a cerebrovascular accident in 29 donors. The CD4+ T helper cellular population was significantly higher in the PBMC of donors. By contrast, CD8+ cytotoxic T cells were highly represented in the backtable fraction of the liver perfusate of matched donors and significantly more abundant compared to CD4+ T cells (*p* < 0.0001) (Figure 1).

**Figure 2:**
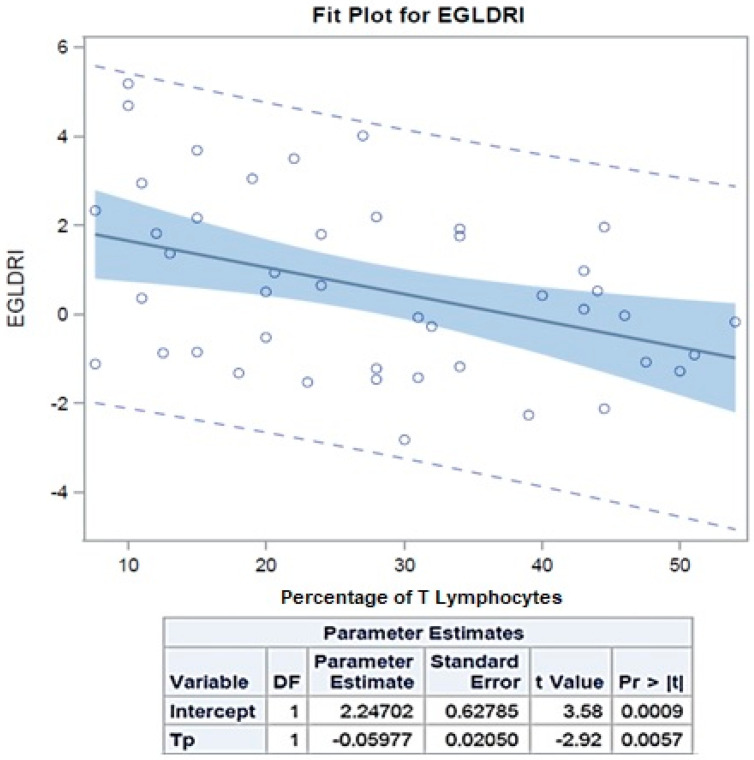
**Linear regression analysis fit plotting percentage of T-cells and EGL-DRI score.** Increasing the percentage of T cells (T-Li) by one reduces the EGL-DRI by 0.05977 (*p*-value = 0.0057).

T-Li percentage was significantly associated with EGL-DRI score (*p* = 0.01), as confirmed by linear regression analysis fit plotting percentage of T-cells and EGL-DRI score (*p*-value = 0.006), and CIT (*p* = 0.02) (Figure 2).

The time in days of DGF (*p* = 0.02) was significantly associated with T-Li percentage, such as the onset of secondary infection from cytomegalovirus (*p* = 0.03). An increased relative risk (HR) of organ survival was significantly associated with the percent cell concentration of T-Li and time to DGF, on COX analysis, were (HR = 1.038, *p* = 0.04; and HR = 1.029, *p* = 0.01, respectively). No relevant clinical outcomes in kidney transplant patients were associated with the specificity of the NK and NK-T cell proportions.

## 4. Discussion

Organ shortage remains a significant challenge in this field of solid organ transplantation. Therefore, it is essential to optimize the use of available organs by ensuring their viability and function [17]. One of the ways to achieve this is by using organ perfusion [18]. The goal of organ perfusion is to maintain the organ viability and function by providing oxygen and nutrients to the cells while removing waste products [19]. This is particularly important in liver and/or kidney transplantation, where the organ is susceptible to ischemia-reperfusion injury, which can lead to primary non-function or DGF. The use of perfusion can reduce the risk of complications and improve the success rate of transplantation, and it might help to prevent the possibility of rejection after transplantation, also being able to hypothesize to identify a category of patients at “low risk of rejection” who can receive lighter immunosuppressive therapy [20].

The most common method of dynamic perfusion in liver transplantation is the use of HMP, which involves cooling the organ to 4 °C and circulating a preservation solution through the blood vessels. HMP has been shown to improve organ function and reduce the risk of complications compared to cold storage [21,22]. Another method of dynamic perfusion is normothermic machine perfusion (NMP), which involves perfusing the organ at body temperature. NMP has been shown to be effective in reducing ischemia-reperfusion injury and improving organ function. NMP can also be used to assess the viability of marginal organs and to repair them ex vivo before transplantation [23]. Both HMP and NMP have been shown to be effective in preserving organ function and reducing the risk of ischemia-reperfusion injury. NMP has the additional advantage of allowing for ex vivo repair of marginal organs. Further research is needed to optimize the use of organ perfusion in liver transplantation and to explore its potential applications in other types of organ transplantation [24]. During the transplantation process, the liver graft is exposed to a variety of pro-inflammatory stimuli, such as ischemia-reperfusion injury, which activates innate immune cells. The following cellular production of pro-inflammatory cytokines and reactive oxygen species leads to tissue damage and inflammation. This process can also affect the renal allograft, which is located near the liver and shares the same blood supply. Activation of the innate immune system can cause early renal allograft injury, which is characterized by acute tubular necrosis and endothelial damage. This can lead to impaired renal function, longer hospital stays, and increased risk of graft loss [25,26].

Several studies have suggested that the presence of certain innate immune cells, such as neutrophils and monocytes, in the peripheral blood can serve as predictive biomarkers of early renal allograft injury. By monitoring these cells before and after transplantation, clinicians may be able to identify patients who are at increased risk of renal injury and provide them with more aggressive monitoring and treatment. The liver contains a large number of immune cells, which maintain the homeostasis between tolerance and inflammation. Liver-resident immune cells infiltrating the sinusoids include professional antigen-presenting cells, myeloid cells, and innate and adaptive lymphocytes. The IRI-associated inflammation can be reduced by machine-perfused organs. However, the role of MP in the function and the activation state of the hepatic immune cells is not clear yet. Theoretically, it remains to be assessed if liver-resident immune cells can induce a pro-inflammatory function on solid organs after machine-perfusion recovery or, on the contrary, mediate liver regeneration and counteract liver damage. However, immune cells resident in the liver have been shown to have a protective anti-inflammatory role for the liver, mediating liver regeneration. Neutrophils, for example, produce cytokines and ROS and perform phagocytosis and proteolysis by the formation of neutrophil extracellular traps (NET) with related angiogenesis. They damage hepatocytes, enhancing local inflammation and promoting graft rejection. By contrast, neutrophils have been shown to participate in tissue regeneration by clearing necrotic debris. Likewise, NK cells have cytotoxic functions and are implicated in tissue damage by releasing pro-inflammatory cytokines like IFN-γ and TNF-α. However, there is also evidence that NK cells also work against biliary epithelial cells. It might contribute to hindering fibrosis by the killing of liver stellate cells. The inhibitory receptor NKG2A can be expressed by a high proportion of hepatic NK cells, and it depletes activated T cells and mediates tolerance induction after transplantation. The liver adaptive immune response is driven by CD4 and CD8 T cells. In the context of MHC I, CD8 T recognizes epitopes from intracellular peptides and produces cytokines, such as IFN-γ and TNF-α. These events can initiate the cytotoxic reaction mediated by further granule releasing the content like granzyme and perforin by triggering Fas-mediated apoptosis. It has been reported that human livers subjected to NMP have reduced numbers of pro-inflammatory cytokines IFN-γ CD8 T cells. CD4 and CD8 T cells are primarily involved in the pathogenesis of IRI. Recently, it was demonstrated that NMP significantly increased the proportion of T-Li in the perfusate throughout the course of perfusion, thus suggesting that donor tissue T-Li is mobilized into the perfusate during perfusion [13].

In deceased donor KT, the proportion of donor T lymphocytes has been suggested to induce the development of DGF, AR, and allograft outcomes. Studies have reported a significant association between a higher proportion of donor T lymphocytes and an increased risk of worse outcomes. The mechanisms underlying this association are not fully understood, but it is hypothesized that the presence of donor T lymphocyte in the transplanted organ may trigger an immune response in the recipient, leading to DGF and AR. It is difficult to speculate on the impact of liver phenotype on graft survival as some immune populations might have a dual controversial impact on tissue integrity. However, it appears clear how the immunological signature plays a fundamental role in the fitness and fate of immune organs, such as the liver, after transplantation. The possibility of mobilizing unwanted immune cells that might pose a risk to graft survival represents a great challenge. Equally important is the method of organ perfusion to guarantee tissue preservation and cellular recovery. Shortage of donations has led to the use of sub-optimal organs, and the identification of the best biomarkers of organ viability and function are the subject of research in many laboratories. The possibility of identifying the immunological signature of liver perfusate to define available organs to be transplanted would improve the utilization of the pool of potential donor organs. In parallel, the identification of detrimental immune factors would permit the development of organ treatment protocols prior to implantation to abrogate ischemia-reperfusion and immunomodulation to prevent rejection [27,28].

Here, we show that by using perfusion of the liver perfusate, it is possible to assess the quality of the organ and identify potential criticisms before transplantation, thereby reducing the risk of complications and improving the success rate of transplantation. Our data are obtained by flow cytometry analysis of the phenotype of liver perfusate from deceased donors. Flow cytometry allows to obtain very rapidly detailed phenotyping of the cellular product from perfusion of the liver. A fast result would permit routine phenotype donors’ livers before organ allocation. Additionally, it would help determine the need for induction therapy, the development of personalized protocols for post-renal transplant immunotherapy, and the monitoring of rejection. However, here we describe a retrospective study that might suffer from selection bias for donor inclusion criteria. Additionally, it is difficult to predict how specific characteristics associated with DBD might influence the homing of donor T cells, thus suggesting that non-specific inflammatory signals related to brain death may alter adaptive immunity.

Some studies have provided insights into how innate immune cells like neutrophils, monocytes, and NK cells were reliable prognostic markers for the detection and monitoring of IRI, clinical complications, and early graft dysfunction [24,25,26,27,28,29,30].

The risk groups of patients based on the quantitative and qualitative phenotyping of the population of CD8 T cells and on the CD4/CD8 ratio is a tool for better risk-adapted monitoring and risk stratification for perioperative complications after KT. In light of this, the proposed methodology could be implemented prospectively in clinical practice and help transplant teams adjust their post-transplant kidney transplant protocols. Furthermore, this approach is particularly important in the context of organ transplantation, where the timely detection of organ damage can be critical for successful transplantation and patient outcomes [30,31]. By using the perfusion fluid of donated organs as a kind of liquid biopsy, clinicians may be able to identify patients who are at increased risk of organ damage following transplantation and provide them with more aggressive monitoring and treatment, which can ultimately improve outcomes for transplant recipients and reduce the risk of graft loss [19,26].

## 5. Conclusions

The proportion of donor T lymphocytes is a potential biomarker for the development of DGF and allograft outcomes following deceased donor kidney transplantation. Studies have reported a significant association between a higher proportion of donor T lymphocytes and an increased risk of DGF and a worse allograft outcome. Further studies are needed to confirm these associations and to investigate them in a prospective setting. Biomarker identification for the development of DGF may help to guide post-transplant management and improve the long-term outcome of KT.

## Figures and Tables

**Table 1:** Donors’ and recipients’ characteristics.

Donors’ Characteristics (n = 42)	
Gender, male (%)	26 (62)
Age (years), ±dev. standard	58.07 ± 14.2
Hypertension (%)	2 (5)
Body mass index (Kg/m^2^), ±dev. standard	25.2 ± 3.9
Type II diabetes mellitus, n (%)	0 (0)
Creatininaemia (mg/dL), ±standard deviation	0.9 ± 0.8
Cause of death	
Cerebro-vascular, n (%)	21 (50)
Trauma, n (%)	11 (26)
Hypoxia, n (%)	8 (19)
Others, n (%)	2 (5)
Infectious risk	
Bacteremia, n (%)	2 (5)
HBcAb+, n (%)	1 (2)
HBsAg+, n (%)	0 (0)
Hepatitis C virus infection, n (%)	1 (2)
HCV and HBcAb + infection, n (%)	0 (0)
Cancer risk, n (%)	2 (5)
**Recipients’ Characteristics (n = 42)**	
Gender, male (%)	27 (59)
Age (years), ±standard dev.	55.06 ± 11.3
Body mass index (Kg/m^2^), ±dev. std.	26.6 ± 3.1
Previous kidney transplant, n (%)	7 (15.2)
Etiology Chronic Renal Failure	
Polycystic kidney	14 (30.4)
Unknown etiology	18 (39.1)
IgA deposits	3 (6.5)
Other	11 (24)
CMV IgG, n (%)	40 (87)
Primary CMV infection, n (%)	5 (10.8)
CMV infection, n (%)	14 (30.4)
New onset diabetes mellitus, n (%)	9 (19.5)
Induction	
Thymoglobulins, n (%)	17 (37)
Basiliximab, n (%)	29 (63)
HLA mismatch	
I level (0–1), n	5
II level (2–4), n	41
III level (5–6), n	0
Acute rejection, n, (%)	10 (21.7)
Graft loss within one year, n, (%)	11 (24)
Delayed graft function, n (%)	23 (50)
Mortality at one year, n (%)	3 (6.5)

**Table 2:** **Deceased brain donor continuous variables**. Comparison of deceased brain donor continuous variables, expressed as the difference between the means of continue variables of the two following populations: inferior and equal or superior to median ranks.

Variable	Mean Diff (1–2)	LowerCLMean	UpperCLMean	StdDev	*p*-Value
HEIGHT (cm)	0.3883	−5.0206	5.7973	9.4085	0.8858
WEIGHT (kg)	4.5683	−3.0132	12.1498	13.1875	0.2315
AGE (years)	−2.3267	−13.6716	9.0183	19.7337	0.6818
BMI (kg/m^2^)	1.3088	−0.5287	3.1464	3.1962	0.1585
ICU STAY (day)	0.4673	−1.2128	2.1474	2.8535	0.5781
Na^+^	−5.4692	−12.8349	1.8965	12.533	0.1418
AST	10.1583	−17.9589	38.2755	48.3464	0.4708
ALT	8	−21.3838	37.3838	49.0093	0.5838
TOT BILIRUBIN	−0.0201	−0.5702	0.5301	0.9615	0.9416
GGT	−3.0227	−53.5032	47.4578	80.8431	0.9043
**EGL-DRI**	**1.4751**	**0.3572**	**2.593**	**1.8353**	**0.0109**
**C.I.T. (min)**	**78.3437**	**6.6644**	**150**	**117.7**	**0.0329**
CD3CD4	−10.6601	−25.758	4.4378	17.7455	0.1580
CD3CD8	8.232	−5.3453	21.8093	15.9582	0.2229
CD56CD3NKG2D	−11.7829	−48.5	24.9343	33.4039	0.5075
**CD56CD3NKp30**	**−25.2182**	**−47.5861**	**−2.8502**	**19.3359**	**0.0298**
CD56CD3NKp44	−3.15	−16.9595	10.6595	15.9714	0.6219
CD56CD3NKp46	4.6236	−29.7991	39.0464	29.7566	0.7775
MAIT (%)	−0.7961	−5.5382	3.946	4.6266	0.7266
NKT (%)	−3.6128	−9.9881	2.7624	11.0892	0.2600
NK/NKT	1.3599	−0.6443	3.3641	3.4861	0.1788
**NK (%)**	**8.9885**	**1.2376**	**16.7394**	**13.482**	**0.0240**

## Data Availability

The data presented in this study are available on request from the corresponding author. The data are not publicly available due to institutional privacy restrictions.

## Abbreviations

ACD: Acid-Citrate-Dextrose; AKI: acute kidney injury; ALT: alanine amino transaminase; AP: alkaline phosphatase; AR: acute rejection; AST: aspartate amino transaminase; CI: confidence interval; CMV: Cytomegalovirus; CTL: cytotoxic T lymphocyte; DAMP: damage-associated molecular pattern; DBD: deceased brain donor; DGF: delayed graft function; DRI: donor risk index; EGL: early graft loss; FBS: Fetal Bovine Serum; GGT: gamma-glutamyl transferase; ICU: intensive care unit; IFN-γ: interferon-gamma; IRI: ischemia-reperfusion injury; IRRB: institutional research review board; KT: kidney transplantation; LP: liver perfusate; NET: neutrophil extracellular traps; NK: Natural Killer; PBMC: peripheral blood mononuclear cells; Th: helper T cells; T-Li: T-lymphocytes; TNF-α: tumor necrosis factor alfa.
